# Mitochondrial oxidative stress promotes atrial fibrillation

**DOI:** 10.1038/srep11427

**Published:** 2015-07-14

**Authors:** Wenjun Xie, Gaetano Santulli, Steven R. Reiken, Qi Yuan, Brent W. Osborne, Bi-Xing Chen, Andrew R. Marks

**Affiliations:** 1 Department of Physiology and Cellular Biophysics, The Wu Center for Molecular Cardiology, and; 2 Department of Medicine, College of Physicians and Surgeons of Columbia University Medical Center, New York, NY, USA

## Abstract

Oxidative stress has been suggested to play a role in the pathogenesis of atrial fibrillation (AF). Indeed, the prevalence of AF increases with age as does oxidative stress. However, the mechanisms linking redox state to AF are not well understood. In this study we identify a link between oxidative stress and aberrant intracellular Ca^2+^ release via the type 2 ryanodine receptor (RyR2) that promotes AF. We show that RyR2 are oxidized in the atria of patients with chronic AF compared with individuals in sinus rhythm. To dissect the molecular mechanism linking RyR2 oxidation to AF we used two murine models harboring RyR2 mutations that cause intracellular Ca^2+^ leak. Mice with intracellular Ca^2+^ leak exhibited increased atrial RyR2 oxidation, mitochondrial dysfunction, reactive oxygen species (ROS) production and AF susceptibility. Both genetic inhibition of mitochondrial ROS production and pharmacological treatment of RyR2 leakage prevented AF. Collectively, our results indicate that alterations of RyR2 and mitochondrial ROS generation form a vicious cycle in the development of AF. Targeting this previously unrecognized mechanism could be useful in developing effective interventions to prevent and treat AF.

Atrial fibrillation (AF), the most common cardiac arrhythmia, has high morbidity and mortality in adults^1^,^2^. Despite intense research for over 100 years, AF remains incompletely understood and treatment is challenging^3^,^4^,^5^,^6^,^7^,^8^. AF can be associated with structural and electrical remodeling of the cardiac atria^4^,^6^,^8^,^9^. Structural changes may directly or indirectly induce atrial electric abnormalities leading to atrial ectopic events and AF. However, it has been difficult to distinguish whether structural changes are the cause or the consequence of AF. In fact, AF has been shown to occur in individuals with structurally normal hearts including those with catecholaminergic polymorphic ventricular tachycardia (CPVT)^10^,^11^ which is linked to inherited mutations in the intracellular Ca^2+^ release channel/ryanodine receptor (RyR2) that cause intracellular Ca^2+^ leak^12^,^13^,^14^. Altered intracellular Ca^2+^ homeostasis has been associated with the pathogenesis of AF. In atrial myocytes, type 2 RyR (RyR2) is the major intracellular Ca^2+^ release channel^14^, and RyR2 dysfunction can affect cellular electric activity. Atrial myocytes from both patients and animals with AF display increased diastolic SR Ca^2+^ leak via RyR2^15^,^16^. Moreover, we and others have reported that AF can be induced in knock-in mice harboring specific mutations in RyR2, leading to intracellular Ca^2+^ leak^17^,^18^,^19^. Recently, a report revealed that AF occurred in a 2-year-old child with a CPVT linked RyR2 mutation^11^. These reports indicate that RyR2 dysfunction may be a critical contributor to AF.

Oxidative stress has been associated with the development of AF both in patients and animal models of AF^20^,^21^,^22^,^23^,^24^, and antioxidant drugs have shown beneficial effects on AF development^21^,^23^,^24^. Furthermore, the prevalence of AF increases with age and the age-dependent increase in oxidative damage is widely acknowledged^25^,^26^. However, the molecular mechanisms underlying oxidative stress in the development of AF remain essentially unclear. RyR2 is an important molecular target of oxidative stress in cardiac myocytes^14^. While stress-induced oxidation of RyR2 in ventricular myocytes has been associated with cardiovascular disease^14^,^27^,^28^,^29^,^30^, little is known about the pathophysiological role of atrial RyR2 oxidation. In a previous report we demonstrated that RyR2 is oxidized in atrial myocytes from a murine model of CPVT, RyR2-R2474S^+/–^ mice, that display substantial increased AF susceptibility^17^.

In the present study, we explored the mechanistic role of atrial RyR2 oxidation in the pathophysiology of AF in two murine models of RyR2-mediated intracellular Ca^2+^ leak: mice harboring an RyR2 mutation linked to human CPVT (RyR2-R2474S^+/–^) and mice expressing a phosphomimetic aspartic acid residue at position 2808 (RyR2-S2808D^+/+^) leading to constitutively leaky channels. Moreover, we evaluated the role of mitochondrial free radicals on RyR2 oxidation by crossing mice harboring RyR2 mutations associated with Ca^2+^ leak with mice overexpressing human catalase targeted to mitochondria (mCAT mice).

## Results

### Atrial RyR2 Oxidation and Leak in Atrial Fibrillation

**Figure 1:**
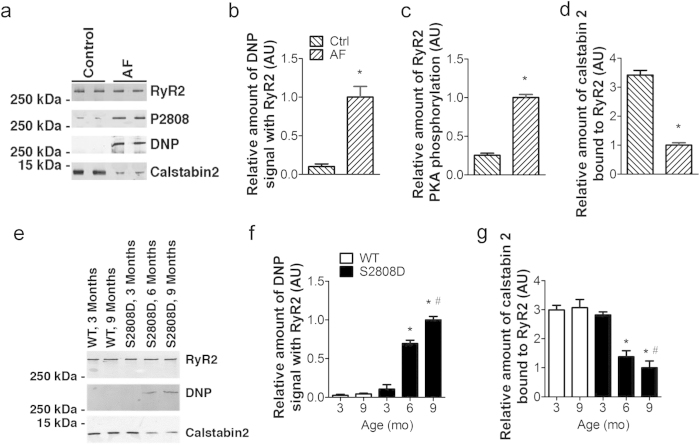
Increased oxidation of the atrial RyR2 complex in patients with AF and in RyR2-S2808D^+/+^ mice. (**a**) post-translational modifications of the RyR2 complex in right atrial (RA) tissue of patients with AF and normal ventricular function or controls. RA appendage tissue was obtained at the time of cardiac surgery from patients with chronic AF (>6 months; n = 10), and patients in sinus rhythm (n = 10). To determine RyR2 channel oxidation, the carbonyl groups in the protein side chains of immunoprecipitated RyR2 were derivatized to (DNP) by reaction with 2,4-dinitrophenylhydrazine. The DNP (2,4-dinitrophenylhydrazone) signal associated with RyR2 was determined by anti-DNP antibody. (**b**–**d**), Quantification of DNP signal (**b**), PKA hyperphosphorylation (**c**), and calstabin 2 bound to RyR2 (**d**) in human atrial samples. *p < 0.01 vs control. Error bars represent s.e.m. (**e**) Post-translational modifications of the RyR2 complex in atrial samples from WT and RyR2-S2808D^+/+^ mice. (**f**) and (**g**), Quantification of DNP signal (f) and calstabin 2 bound to RyR2 (**g**); atrial samples were obtained from at least 5 mice in each group. AU: arbitrary units. All data are shown as mean  ±  s.e.m. * and **: p < 0.05 and 0.01 vs 3-month-old group; ^#^: p < 0.05 vs WT.

Atrial RyR2s from patients with chronic AF were oxidized, phosphorylated and depleted of calstabin 2 (a subunit of the complex that stabilizes the closed state of RyR2 during diastole^14^,^31^) compared to subjects in sinus rhythm (Fig. 1a–d). These results are consistent with our previous report in mice harboring CPVT-mutated RyR2 channels, where RyR2 channels were oxidized and DTT treatment reduced SR Ca^2+^ leak in atrial myocytes^17^.

**Figure 2:**
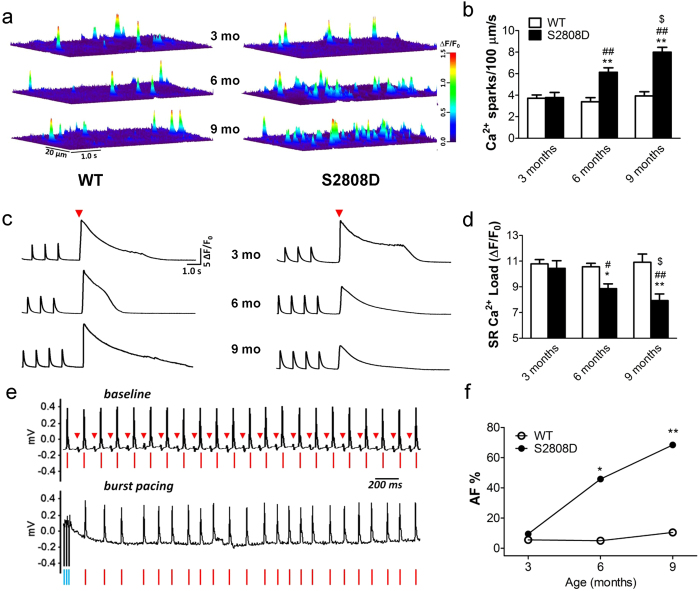
Age-dependent increases in SR Ca^2+^ leak in atrial myocytes correlate with AF susceptibility in RyR2-S2808D^+/+^ mice. (**a**) Representative Ca^2+^ sparks images in atrial myocytes from WT and RyR2-S2808D^+/+^ mice. (**b**) Ca^2+^ spark frequencies in atrial myocytes from WT and RyR2-S2808D^+/+^ mice with the indicated ages, n = 50~60 cells from 3 ~ 4 mice in each group. (**c**) Representative trace of caffeine-induced (10 mM, red triangles) Ca^2+^ transients immediately following 1-Hz field stimulation in atrial myocytes from WT (left) and RyR2-S2808D^+/+^ (right) mice; ‘mo’ = months. (**d**) SR Ca^2+^ load in atrial myocytes from WT and RyR2-S2808D^+/+^ mice at the indicated ages, n = 25 ~ 28 cells from 3 ~ 4 mice in each group. (**e**) Representative surface ECG traces of intra-esophageal burst pacing-induced AF in 9-month-old RyR2-S2808D^+/+^ mice. Upper panel shows ECG trace before pacing and lower panel shows ECG following termination of burst pacing. Red triangles indicate P waves and red lines indicate QRS complexes, consistent with normal sinus rhythm. Lower panel shows irregular rhythm; blue lines indicate burst pacing currents. (**f**) Prevalence of AF during intra-esophageal burst pacing in WT and RyR2-S2808D^+/+^ mice at different ages. All data are shown as mean ± s.e.m. * and **: p < 0.05 and 0.01 vs WT; ^#^, ^##^: p < 0.05 and 0.01 vs 3-month-old; ^$^: p < 0.05 vs 6-month-old.

To explore the role of intracellular Ca^2+^ leak via RyR2 in the development of AF, we used a transgenic mouse harboring a constitutively leaky RyR2 channel (RyR2-S2808D^+/+^), which displays an age-related RyR2 oxidation in ventricular myocytes^32^. WT and RyR2-S2808D^+/+^ littermates were divided into three different age groups (3-, 6- and 9-month old). Similar to ventricular RyR2, atrial RyR2 from RyR2-S2808D^+/+^ mice exhibited age-related oxidation and depletion of calstabin 2 compared to RyR2 from WT mice which displayed no PKA phosphorylation of RyR2 at 3-, 6- and 9-months of age (Fig. 1e–g, Supplementary Figure 1). To measure intracellular Ca^2+^ leak and its effects we examined Ca^2+^ spark frequency and SR Ca^2+^ load. At 3 months of age there was no significant difference in Ca^2+^ spark frequency (Ca^2+^ sparks/100 μm/s: 3.80 ± 0.47 in S2808D *vs*. 3.69 ± 0.31 in WT, n.s.) and SR Ca^2+^ load (∆F/F_0_: 1_0_.43 ± 0.59 in S2808D *vs*. 10.78 ± 0.33 in WT, n.s.) in atrial myocytes isolated from WT versus RyR2-S2808D^+/+^ mice (Fig. 2a–d). In contrast, at 6 months of age atrial myocytes from RyR2-S2808D^+/+^ mice displayed significantly higher Ca^2+^ spark frequencies (Ca^2+^ sparks/100 μm/s: 6.13 ± 0.42 *vs*. 3.39 ± 0.38 in WT, p < 0.01) and markedly depleted SR Ca^2+^ stores (∆F/F_0_: 8.86 ± 0.37 *vs*. 10.56 ± 0.27 in WT, p < 0.05), consistent with age-related intracellular Ca^2+^ leak, compared with WT littermates (Fig. 2a–d). These differences were further increased in 9-month-old mice (Ca^2+^ sparks/100 μm/s: 8.00 ± 0.45 in S2808D *vs*. 3.93 ± 0.40 in WT, p < 0.01; SR Ca^2+^ load (∆F/F_0_: 7.93 ± 0.51 in S2808D *vs*. 10.93 ± 0.64 in WT, p < 0.01) as shown in Fig. 2A–D. Despite the observed depletion of SR Ca^2+^ stores, there was no significant change in the paced Ca^2+^ transient amplitude (Supplementary Figure 2a). This is most likely attributable to the increased fractional release of Ca^2+^ (Supplementary Figure 2b) due to a leftward shift in the Ca^2+–^sensitivity to activation of RyR2. Consistent with the increased SR Ca^2+^ leak in atrial myocytes, *ex vivo* confocal imaging in intact hearts^18^,^33^ and *in vitro* confocal imaging in isolated atrial myocytes revealed increased intracellular arrhythmic Ca^2+^ activity in the atria of 9-month-old RyR2-S2808D^+/+^ mice compared to WT littermates (Supplementary Figures 3–4). Similar to RyR2-R2474S^+/–^ mice^17^, the SR Ca^2+^ leak observed in atrial myocytes isolated from 9-month-old RyR2-S2808D^+/+^ mice was reduced by either 2-hour treatment with the Rycal S107 (10 μM), which prevented the oxidation-induced dissociation of calstabin 2 from RyR2, or treatment with the reducing agent dithiothreitol (DTT, Supplementary Figure 5a). CaMKII-mediated RyR2 phosphorylation has been recently reported to be involved in AF^34^. However, KN-93 (1 μM), a CaMK inhibitor^35^, did not significantly reduce the SR Ca^2+^ leak in atrial myocytes from RyR2-S2808D^+/+^ and RyR2-R2474S^+/–^ mice (Supplementary Figure 5).

### Age-dependent increase of AF susceptibility in RyR2-S2808D^+/+^ mice

AF can be linked to intracellular arrhythmic Ca^2+^ activity in atrial myocytes^18^. To investigate AF susceptibility in RyR2-S2808D^+/+^ mice we induced AF by pacing the left atrium via an endo-esophageal pacing catheter. At baseline, all mice were in normal sinus rhythm (Fig. 2e). Upon pacing, RyR2-S2808D^+/+^ mice exhibited a progressive, age-related augmented susceptibility to AF in response to atrial burst pacing measured as the percentage of mice with pacing induced AF: 9.7% (3/31) at 3 months; 45.8% (11/24) at 6 months; and 68.4% (13/19) at 9 months). In contrast AF was rarely inducible in WT mice at the same ages: 5.6% (2/36); 5.0% (1/20); and 10.5% (2/19) at 3, 6 and 9 months of age, respectively (Fig. 2f).

AF has also been associated with structural remodeling of the atria^4^,^6^,^7^,^9^. However, histological analyses of atrial tissue did not show any obvious structural abnormality in 9-month-old RyR2-S2808D^+/+^ mice (Supplementary Figure 6), suggesting that the altered Ca^2+^ homeostasis is the main factor contributing to AF in this mouse model. Moreover, a 2-week pharmacological treatment with a stabilizer^14^,^18^ of the closed state of RyR2 channel (S107, 40 mg/kg/d in drinking water) prevented the development of AF in 9-month-old RyR2-S2808D^+/+^ mice (Supplementary Figure 7).

### Increased intracellular oxidative stress in atrial myocytes from RyR2-S2808D^+/+^ mice

**Figure 3:**
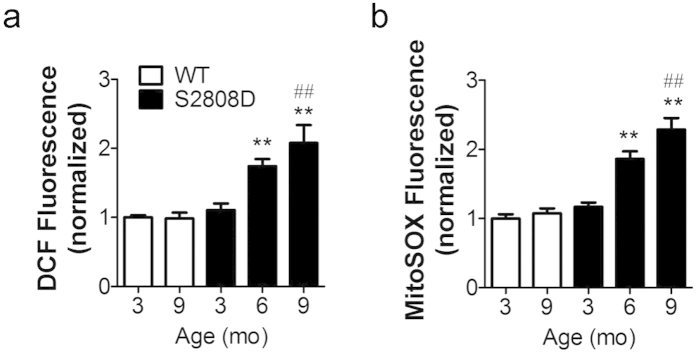
Increased intracellular oxidative stress and mitochondrial ROS levels in WT and RyR2-S2808D^+/+^ mice. (**a**) atrial intracellular oxidative stress measured by CM-H_2_DCFDA, n > 100 cells from 3 ~ 4 mice in each group. (**b**) mitochondrial ROS levels in atrial myocytes, n > 140 cells from 3 ~ 4 mice in each group. The DCF and mitoSOX fluorescence are normalized to fold of 3-month-old WT group. All data are shown as mean ± s.e.m. * and **: p < 0.05 and 0.01 vs 3-month-old group; ^#^, ^##^: p < 0.05 and 0.01 vs WT.

**Figure 4:**
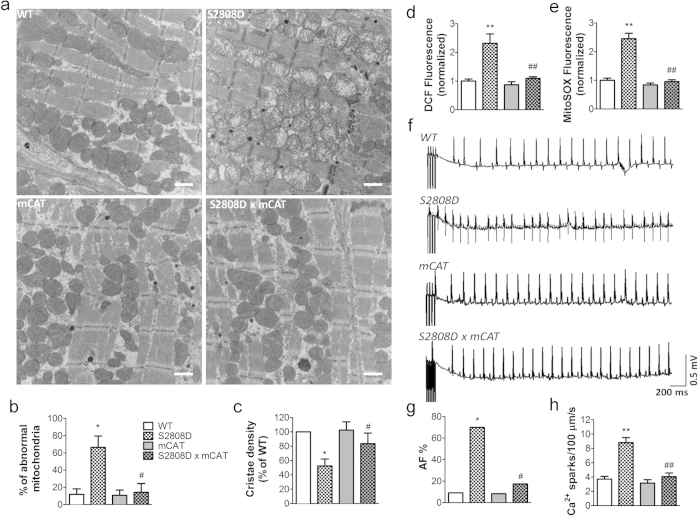
Genetic reduction of mitochondrial ROS levels prevents mitochondrial dysmorphology, atrial SR Ca^2+^ leak and AF in 9-month-old RyR2-S2808D^+/+^ mice. (**a**) Representative transmission electron micrographs of atrial mitochondria from WT, RyR2-S2808D^+/+^, mCAT, and RyR2-S2808D^+/+^/mCAT^+^ mice. n = 3/group, 20000 × magnification, *scale bar: 1* *μm*. (**b**) and (**c**) Morphometric analysis of atrial mitochondria reveals abnormalities in RyR2-S2808D mice. Abnormal mitochondria were defined by the loss of electron density in more than 20% of the area of a mitochondrion. The relative number of damaged mitochondria was quantified by blinded observers from at least 20 images from different fields of atrial samples. The dense core granules represent the atrial specific granules, considered the storage site of atrial natriuretic peptide. (**d**) and (**e**) Assessment of intracellular oxidative stress (**d**) and mitochondrial ROS level (**e**) in atrial myocytes. n > 100 cells from 3 mice in each group, the DCF and mitoSOX fluorescence are normalized to fold of WT group. (**f**) Representative surface ECG traces from indicated mice during intra-esophageal burst pacing. (**g**) Prevalence of AF in WT (n = 11), RyR2-S2808D^+/+^ (n = 11), mCAT (n = 12), and RyR2-S2808D^+/+^/mCAT^+^ (n = 23) mice. (**h**) Ca^2+^ sparks frequencies in atrial myocytes isolated from mice in these groups, n = 40 ~ 47 cells from at least 3 mice in each group. All mice were 9-months-old. All data are shown as mean ± s.e.m. * and **: p < 0.05 and 0.01 vs WT; ^#^, ^##^: p < 0.05 and 0.01 vs RyR2-S2808D^+/+^.

Reactive oxygen species (ROS) production measured using the fluorescent indicator H_2_DCFDA was significantly increased in freshly isolated atrial myocytes from 6- and 9- month old RyR2-S2808D^+/+^ mice, indicating increased oxidative stress (Fig. 3a). Mitochondria are the major source of intracellular ROS^36^,^37^. MitoSOX Red revealed that atrial myocytes from 6- and 9-month old RyR2-S2808D^+/+^ mice had significantly higher mitochondrial ROS level (Fig. 3b). Additionally, ultrastructural analyses revealed profound mitochondrial dysmorphology, including lamellar degeneration, outer membrane disruption and swelling, in atria from 9-month old RyR2-S2808D^+/+^ mice, compared with age-matched WT littermates (Fig. 4a–c).

### Genetic inhibition of mitochondrial ROS production reduces AF

To further investigate the role of mitochondrial ROS in atrial RyR2 dysfunction we crossed RyR2-S2808D^+/+^ mice with transgenic mice (mCAT) overexpressing the human catalase gene targeted to mitochondria to decrease mitochondrial ROS production. RyR2-S2808D^+/+^/mCAT mice exhibited significantly reduced atrial mitochondrial abnormalities (Fig. 4a–c). In atrial myocytes isolated from 9-month old RyR2-S2808D^+/+^/mCAT mice, both cellular and mitochondrial oxidative stress were markedly reduced compared with age-matched RyR2-S2808D^+/+^ littermates (Fig. 4d–e). Strikingly, blunted ROS production was associated with reduced atrial RyR2 oxidation and castabin2 dissociation resulting in decreased atrial diastolic SR Ca^2+^ leak and AF susceptibility (Fig. 4f–h and Supplementary Figure 8-9).

### S107 improves mitochondrial function

**Figure 5:**
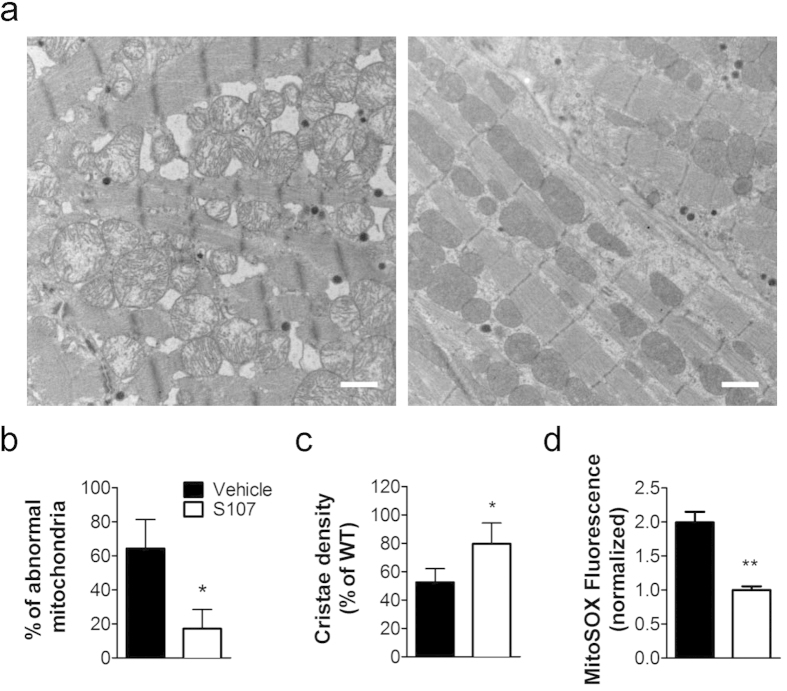
Inhibition of SR Ca^2+^ leak attenuates mitochondrial ROS production and dysfunction. (**a**) Representative transmission electron micrographs of atrial mitochondria from 9-month-old RyR2-S2808D^+/+^ mice with 2-month S107 or vehicle treatment. n = 3/group, 20000 × magnification, *scale bar: 1* *μm*. (**b**) and (**c**) Morphometric analyses of atrial mitochondria show attenuated abnormalities in S107 treated group. (**d**) mitochondrial ROS levels in atrial myocytes from 9-month-old RyR2-S2808D^+/+^ mice following a 2-month treatment with S107 or vehicle. n > 100 cells from each group, the mitoSOX fluorescence are normalized to fold of S107 group. All data are shown as mean ± s.e.m. * and **: p < 0.05 and 0.01 vs vehicle.

To test the hypothesis that atrial mitochondrial dysfunction is attributable to SR Ca^2+^ leak, we examined the effects of S107 treatment on atrial mitochondria of RyR2-S2808D^+/+^ mice. Following a 2-month treatment with S107 (40 mg/kg/d in the drinking water), both mitochondrial dysmorphology (Fig. 5a–c) and ROS production (Fig. 5d) were significantly reduced in 9-month-old RyR2-S2808D^+/+^ mice compared with vehicle group.

### Genetic inhibition of mitochondrial ROS prevents AF

**Figure 6:**
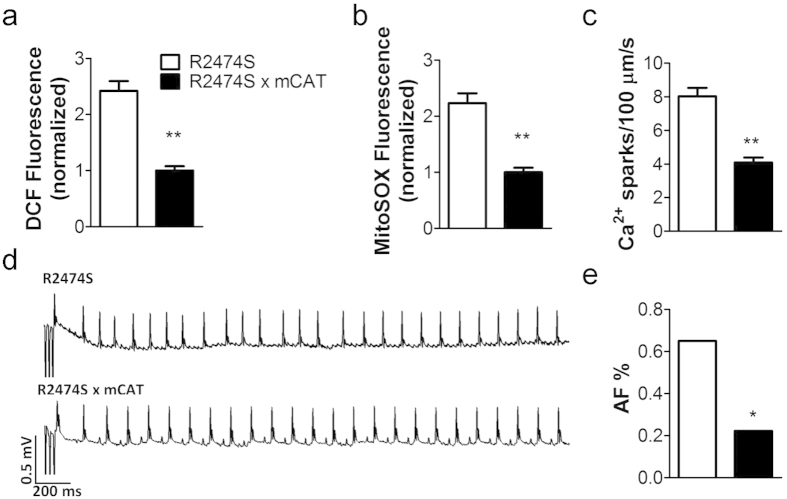
Genetic reduction of mitochondrial ROS levels prevents atrial SR Ca^2+^ leak and AF in RyR2-R2474S^+/–^ mice. (**a–c**), mitochondrial ROS levels (**a**), intracellular oxidative stress (**b**) and SR Ca^2+^ leak (**c**) in atrial myocytes from 3-month-old RyR2-R2474S^+/–^ and RyR2-R2474S^+/–^/mCAT mice. For A and B, n > 100 cells from each group, the DCF and mitoSOX fluorescence are normalized to fold of R2474S x mCAT group. For C, n = 40 cells from 5 mice in each group. (**d**) Representative surface ECG traces from indicated mice following intra-esophageal burst pacing. (**e**) Prevalence of AF in RyR2-R2474S^+/–^ (n = 20) and RyR2-R2474S^+/–^/mCAT^+^ (n = 18) mice. All data are shown as mean ± s.e.m. * and **: p < 0.05 and 0.01.

RyR2-R2474S^+/–^ mice display oxidized atrial RyR2 and AF susceptibility^17^ and increased mitochondrial ROS level (Supplementary Figure 10). Both mitochondrial ROS levels and intracellular oxidative stress were greatly reduced in RyR2-R2474S^+/–^/mCAT mice compared with RyR2-R2474S^+/–^ mice (Fig. 6a–b). Similarly, crossing RyR2-R2474S^+/–^ with mCAT mice significantly decreased the intracellular Ca^2+^ leak in atrial myocytes compared to RyR2-R2474S^+/–^ mice (Fig. 6c). Importantly, AF susceptibility in RyR2-R2474S^+/–^/mCAT was diminished to 22.2% (4/18) compared with 65.0% (13/20) in RyR2-R2474S^+/–^ mice (Fig. 6d–e).

## Discussion

Accumulating evidence suggests that oxidative stress plays a pivotal role in the development and perpetuation of AF^20^,^21^,^22^,^38^,^39^. Our present findings strongly support previous reports suggesting that AF is associated with myocardial oxidative stress^20^,^38^.

We and others have proposed that atrial diastolic SR Ca^2+^ leak is an essential contributing factor in the pathogenesis of AF^17^,^18^,^19^. Diastolic SR Ca^2+^ leak due to RyR2 dysfunction may be linked to multiple factors including phosphorylation, oxidation or pathological mutations of RyR2, all of which can result in channel dysfunction^14^. We have previously reported PKA hyperphosphorylation and calstabin2 dissociation from RyR2 in several models of chronic AF^15^. We have also demonstrated that chronic PKA phosphorylation of ventricular RyR2 is associated with oxidation of the channel and the combination of PKA phosphorylation and oxidation of RyR2 results in significantly more calstabin 2 dissociation from RyR2 compared to each post-translational modification alone^27^. This observation is consistent with our present results, indicating the importance of oxidation of atrial RyR2 in promoting AF. Furthermore, oxidation of atrial RyR2 has been shown to be a key contributor to diastolic SR Ca^2+^ leak and AF in animal models of CPVT^17^.

It has been reported that CaMKII is also a molecular target of oxidative stress, and that oxidized CaMKII can phosphorylate RyR2 at Ser^2814^ inducing intracellular Ca^2+^ leak^40^. However, we found that KN-93 treatment did not reduce atrial SR Ca^2+^ leak in RyR2-S2808D^+/+^ or RyR2-R2474S^+/−^ atrial cardiomyocytes, suggesting that direct oxidation of atrial RyR2, but not phosphorylation by oxidized CaMKII, is the main factor inducing atrial SR Ca^2+^ leak and AF.

The importance of PKA phosphorylation in cardiac disease has been challenged by Valdivia’s group, who concluded that phosphorylation of Ser^2808^ plays no role in β-adrenergic cardiac response. The fact that our findings have been confirmed by multiple other groups is addressed in a recent review^32^.

Multiple sources of ROS, including mitochondria, NADPH oxidases and NOS uncoupling, contribute to AF^5^,^8^,^41^. Intriguingly, recent reports indicate that atrial sources of ROS vary with the duration and the substrate of AF^42^, and mitochondria have been proposed as the major ROS source for long-term AF and age-related functional decline^25^. This view is consistent with our results showing that mitochondrial ROS promote age-related AF.

In the present study we establish for the first time that atrial RyR2 is a specific molecular target of oxidative stress that is fundamental in the development of AF. We demonstrate the functional importance of RyR2 oxidation in AF pathophysiology, showing that mitochondrial-derived ROS oxidize RyR2 in atrial myocytes leading to increased intracellular Ca^2+^ leak. Importantly, reducing mitochondrial ROS production attenuates atrial diastolic SR Ca^2+^ leak and prevents AF.

Mitochondria have been reported to be abnormal in the atrial tissue of patients with AF^39^,^43^. In this study, mice harboring leaky RyR2 also display mitochondrial abnormalities and increased ROS production. In cardiac myocytes, mitochondria and the SR are co-localized in the ‘mitochondrial microdomain^44^’. Since mitochondrial Ca^2+^ uptake via the mitochondrial Ca^2+^ uniporter is dependent on SR Ca^2+^ release^44^,^45^,^46^, alterations in SR Ca^2+^ release can also affect mitochondrial function by regulating mitochondrial Ca^2+^ uptake^44^,^47^. In atrial myocytes isolated from RyR2-S2808D^+/+^ mice, leaky RyR2 channels were associated with an age-related increase in diastolic SR Ca^2+^ release without any significant change in the systolic Ca^2+^ transient amplitudes. Pharmacological inhibition of RyR2 Ca^2+^ leak restored atrial mitochondrial morphology and function suggesting that mitochondrial Ca^2+^ overload plays a key role in AF pathophysiology.

Taken together, our data demonstrate that RyR2 oxidation resulting from intracellular oxidative stress in atrial myocytes leads to increased SR Ca^2+^ leak contributing to the pathogenesis of AF. Alterations of RyR2 inducing intracellular Ca^2+^ leak, including constitutive PKA phosphorylation or CPVT mutations, trigger a vicious cycle, in which SR Ca^2+^ leak in atrial myocytes impairs mitochondrial function leading to an increase in ROS production, thereby promoting RyR2 oxidation and further Ca^2+^ leak. Pharmacological targeting of leaky RyR2 channels or genetically inhibiting mitochondrial ROS production prevents AF providing mechanistic insights that could lead to new therapeutic targets for AF.

## Methods

### Human studies

All human analyses were performed in accordance with protocols approved by the Institutional Review Board of the New York Presbyterian Hospital and by the Ethics Committee of Columbia University. Written informed consent was obtained from all participants. RA appendage tissue was obtained at the time of cardiac surgery from patients with chronic AF (>6 months; n = 10), and patients in sinus rhythm (n = 10).

### Animal studies

All animal experiments were performed in accordance with NIH Guidelines for the Care and Use of Laboratory Animals, and animal protocols were approved by the Institutional Animal Care and Use Committee (IACUC) of Columbia University.

Generation of the mCAT mouse has been described previously^48^. RyR2-R2474S^+/-^ and RyR2-S2808D^+/+^ mice were generated as described^17^,^45^. All mice were backcrossed into the C57BL/6 background for >10 generations. All *in vivo* and *in vitro* experiments were conducted by operators who were blinded to the genotypes of the mice.

### S107 treatment

For *in vitro* experiments using isolated atrial myocytes, 10 μM S107 was added to the extracellular solution for 2 hours. For *in vivo* experiments, S107 was diluted in drinking water at 0.25 mg/ml (40 mg/kg/d). No differences were detected in water consumption between vehicle and S107-treated groups.

### Intra-esophageal burst pacing in mouse

Intra-esophageal pacing was performed by placing in the esophagus, close to the left atrium, a 1.1-Fr octapolar catheter (EPR-800, Millar Instruments, Houston, Texas) connected to an external stimulator (STG-3008, MultiChannel Systems, Reutlingen, Germany). A computerized data acquisition system (EMKA Technologies, Falls Church, VA) was used to record a 3-lead surface ECG, and up to 4 intra-esophageal bipolar electrocardiograms. Inducibility of atrial arrhythmias was tested by applying a series of 2-second bursts. The first 2-second burst had a cycle length (CL) of 40 ms; then the CL was progressively decreased by 2 ms in each successive burst until reaching 10 ms. AF was defined as a period of rapid irregular atrial rhythm lasting at least 1 sec.

### Isolation of adult murine atrial myocytes

Adult murine atrial myocytes were isolated as follows. The heart was rapidly isolated, cannulated and perfused with AfCS perfusion buffer, comprised of (in mM): NaCl 113, KCl 4.7, KH_2_PO_4_ 0.6, Na_2_HPO_4_ 0.6, MgSO_4_ 1.2, NaHCO_3_ 12, KHCO_3_ 10, HEPES 10, taurine 30, glucose 1.5 and 2,3-Butanedione 2-monoxime (BDM) 10, for 5 minutes at a speed of 3 ml/min. Then, perfusion was switched to ‘digestion buffer’ (which includes 0.65 mg/mL type 2 collagenase and 50 μM CaCl_2_ in AfCS) and perfused for 10–15 minutes. Atria were cut and teased into small pieces in ‘stop 1’ buffer (0.65 mg/mL type 2 collagenase, 0.065 mg/mL Protease XIV, 15 mg/mL BSA and 50 μM CaCl_2_ in AfCS) and bathed at 37 °C for 10 minutes after which the enzyme was removed by centrifugation (4 min at 200 rpm). The cells were then resuspended in ‘stop 2’ buffer (15 mg/mL bovine serum albumin and 50 μM CaCl_2_ in AfCS) and gradually recovered to a final [Ca^2+^] of 1.8 mM.

### Ca^2+^ imaging in isolated atrial myocytes

To measure intracellular Ca^2+^, cells were loaded with 5 μM Fluo-4 AM (ThermoFisher Scientific, Waltham, MA) for 20 minutes and washed three times and maintained in the following solution (in mM): NaCl 125, KCl 4.75, MgSO_4_ 1.2, KH_2_PO_4_ 1.2, HEPES 30, glucose 10, taurine 50, CaCl_2_ 2 and pH = 7.4. A Leica TCS SP2 confocal microscopy with 40x, 1.25 NA oil immersion objectives was used for linescan imaging. The scan zoom was adjusted to fit the cells, and scan line was performed along the long axis of cells^49^. The excitation for Fluo-4 was 488 nm, while emission was collected at 505–530 nm. For Ca^2+^ sparks recording, cells were scanned at 400 Hz for 20 s following 1 minute of pacing at 3 Hz. Ca^2+^ sparks detection and analysis was performed as previously described^49^,^50^,^51^. KN-93 was purchased from Sigma-Aldrich (St. Louis, MO). For simultaneous recording of Ca^2+^ transient amplitudes and SR Ca^2+^ contents, cells were exposed to 10 mM caffeine immediately following termination of pacing at 1 Hz for 1 minute. Sampling started 10 s before caffeine treatment.

### Measurements of intracellular oxidative stress

For the evaluation of intracellular oxidative stress, cells were pre-incubated with the chloromethyl derivative CM-H_2_DCFDA (10 μM, ThermoFisher Scientific) for 30 minutes and washed. Fluorescence intensity and images were obtained using a confocal microscope (Zeiss 5 Live, 40 x oil immersion lens). Excitation was at 488 nm, and emission was collected at 505–530 nm. Since CM-H_2_DCFDA is light sensitive and oxidized progressively, we used the same scanning parameters for all experiments. For each dish, images were rapidly acquired for ~10 randomly selected cells. More than 100 cells per group were examined for intracellular fluorescent intensities.

### Mitochondrial ROS detection

For mitochondrial ROS detection, cells were incubated for 20 min with 5 μM MitoSOX Red (Invitrogen/Molecular probes) and washed. Fluorescence intensity and images were obtained using confocal microscopy (Zeiss 5 Live, 40x oil immersion lens). MitoSOX Red was excited at 488 nm and emission was collected at 540–625 nm. The scanning parameters were unchanged for all the scans. For each group, fluorescence intensities of >100 cells randomly selected from several different dishes were examined.

### Immunoprecipitation and immunoblotting analysis of RyR modifications

RyR2 was immunoprecipitated from atrial homogenates (250 μg) using anti-RyR antibody in 0.5 ml of a modified RIPA buffer (50 mM Tris-HCl pH 7.4, 0.9% NaCl, 5.0 mM NaF, 1.0 mM Na_3_VO_4_, 0.5% Triton-X100, and protease inhibitors) overnight at 4 °C. The samples were incubated with protein A sepharose beads (Amersham Pharmacia Biotech, Piscataway, NJ) at 4 °C for 1 hr and washed five times with 1.0 ml RIPA. Samples were heated to 95 °C and size fractionated by PAGE (6% for RyR2, 15% for calstabin 2). Levels of RyR2 bound proteins were normalized to the total RyR2 immunoprecipitated. All immunoblots were developed using the Odyssey system (LI-COR, Inc., Lincoln, NE) with IR labeled anti-mouse and anti-Rabbit IgG (1:10000 dilution) secondary antibodies^52^. To detect RyR2 protein oxidation, atrial SR membrane samples (50 ug) were immunoprecipitated as described above. Immunoprecipitates were treated with 2, 4-dinitrophenylhydrazine (DNP) and the derivatized carbonyls were detected using an OxyBlot^TM^ Protein Oxidation Detection Kit (Cat # S7150, Chemicon International, Inc., Temecula, CA)^45^,^49^. Proteins were size fractionated on 6% SDS-PAGE gels and transferred onto nitrocellulose membranes and immunoblots were developed with an anti-RyR antibody (Affinity Bioreagents, Bolder, CO, 1:2,000). The DNP signal associated with RyR was determined using an anti-DNP antibody (1:2000)^45^.

### Transmission Electron Microscopy

Atria were fixed in 2.5% glutaraldehyde in 0.1 M Sørensen’s buffer and post-fixed in 1% OsO_4_. Following dehydration, samples were embedded in Lx-112 (Ladd Research Industries, Williston, VT). After cutting (ultramicrotome MT-7000), the 60 nm sections were stained with uranyl-acetate and lead-citrate and visualized (JEM-1200 EXII, JEOL, Tokyo, Japan), as previously described^45^,^49^. For each animal at least twenty randomly selected sections were used for the analysis of mitochondrial morphology.

### Statistical analysis

All results are presented as mean  ±  s.e.m. Statistical analyses were performed using the unpaired Student’s *t* test (for 2 groups) and one-way ANOVA with Bonferroni *post hoc* correction (for groups of 3 or more) unless otherwise indicated. *P* < 0.05 was considered to be statistically significant.

## Additional Information

**How to cite this article**: Xie, W. *et al.* Mitochondrial oxidative stress promotes atrial fibrillation. *Sci. Rep.* **5**, 11427; doi: 10.1038/srep11427 (2015).

## Supplementary Material

Supplementary Information
