# Supplementary material for: Mitochondrial oxidative stress promotes atrial fibrillation
Source: Sci Rep. 2015 Jul 14;5:11427. doi: 10.1038/srep11427 (PMC4501003; doi:10.1038/srep11427)
Supplement: Supplementary Information [file srep11427-s1.doc]

**Mitochondrial oxidative stress promotes atrial fibrillation**

Wenjun Xie^1*^, Gaetano Santulli^1*^, Steven R. Reiken^1^, Qi Yuan^1^, Brent W. Osborne^1^, Bi-Xing Chen^1^, Andrew R. Marks^1,2^

*Wenjun Xie and Gaetano Santulli contributed equally to this work.

**SUPPLEMENTAL FIGURES and LEGENDS**


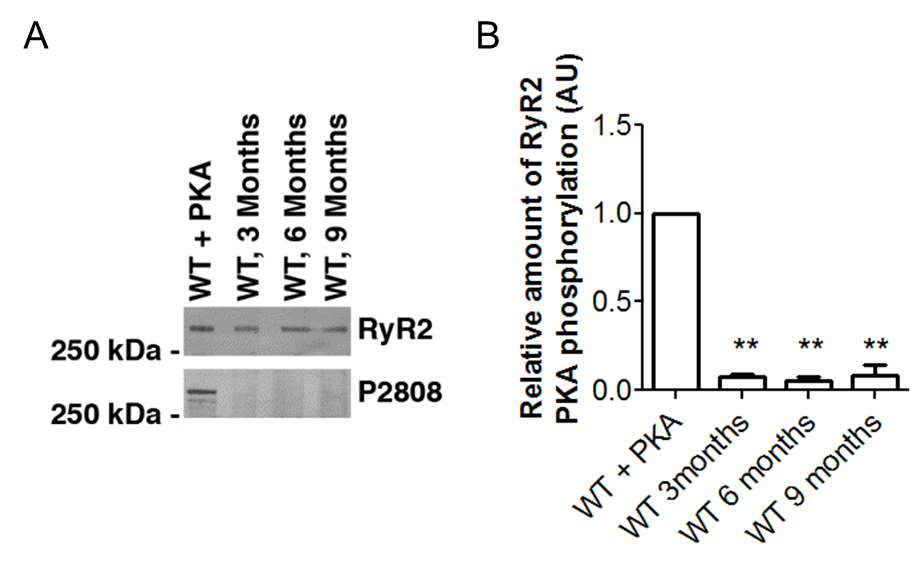


**Supplementary Figure 1**. **Lack of PKA phosphorylation of atrial RyR2** **in control WT mice at 3, 6 and 9 months of age** **(A)** compared with WT PKA treated samples as control. (B) Quantification of data represented in (A). Atrial samples were obtained from at least 5 mice in each group. Data are shown as mean ± s.e.m. **, p<0.01 vs WT+PKA.


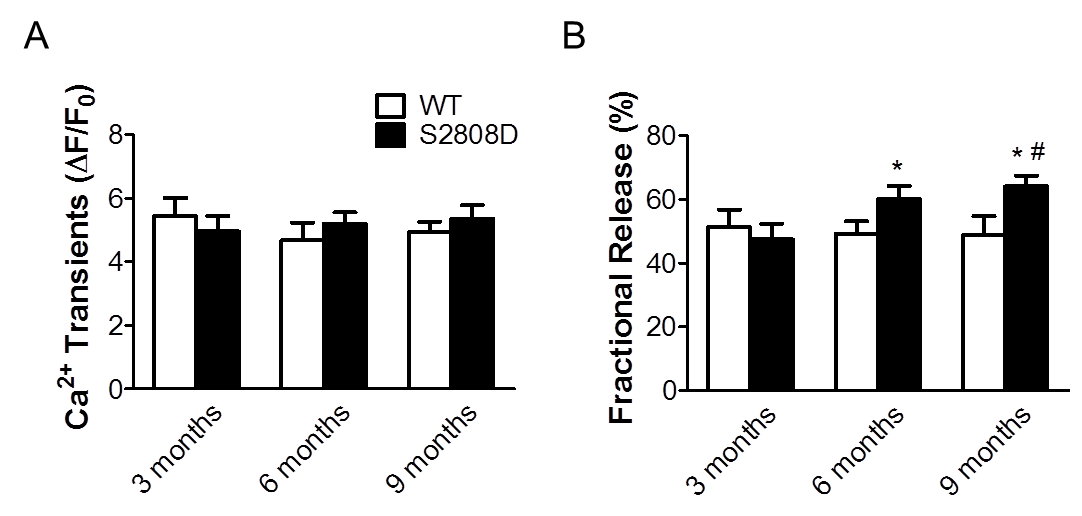


Supplementary Figure 2. Increased fractional release of SR Ca^2+^ in RyR2-S2808D cardiac myocytes indicating that chronic PKA phosphorylation of RyR2 can result in maintained SR Ca^2+^ release despite reduced SR Ca load due to increased fractional release of SR Ca^2+^ due to leaftward shift in the sensitivity of cytosolic Ca^2+^ induced activation of RyR2. Ca^2+^ transients (A) amplitude and SR fractional Ca^2+^ release (B) in atrial myocytes from WT and RyR2-S2808D^+/+^ mice at the indicated ages. n=20~28 cells from ≥3 mice in each groups. Data are shown as mean ± s.e.m. *, p<0.05 vs WT; ^#^, p<0.05 vs 3-month-old.


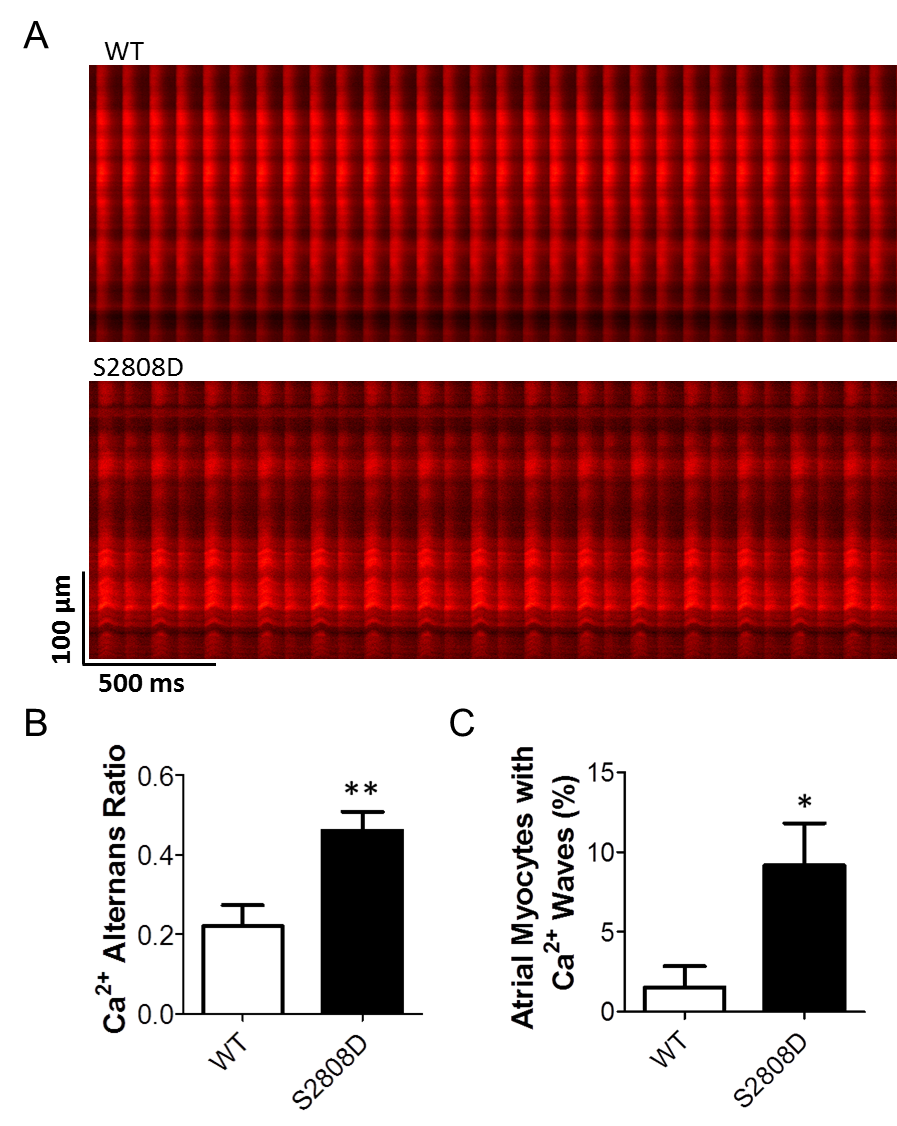


Supplementary Figure 3. Increased atrial Ca^2+^ alternans and Ca^2+^ waves in intact hearts from 9-month-old RyR2-S2808D^+/+^ mice compared to WT controls.

**A**, Representative images of 10 Hz pacing-induced Ca^2+^ alternans in right atrial tissue from 9-month-old WT and RyR2-S2808D^+/+^ mice. **B**, Quantification of atrial Ca^2+^ alternans in the indicated groups. **C**, Atrial Ca^2+^ waves in the indicated groups. Hearts were perfused with 4 mM [Ca^2+^] to induce Ca^2+^ waves. n=at least 6 hearts in each group. All Data are shown as mean ± s.e.m. * and **, p<0.05 and 0.01 vs WT.


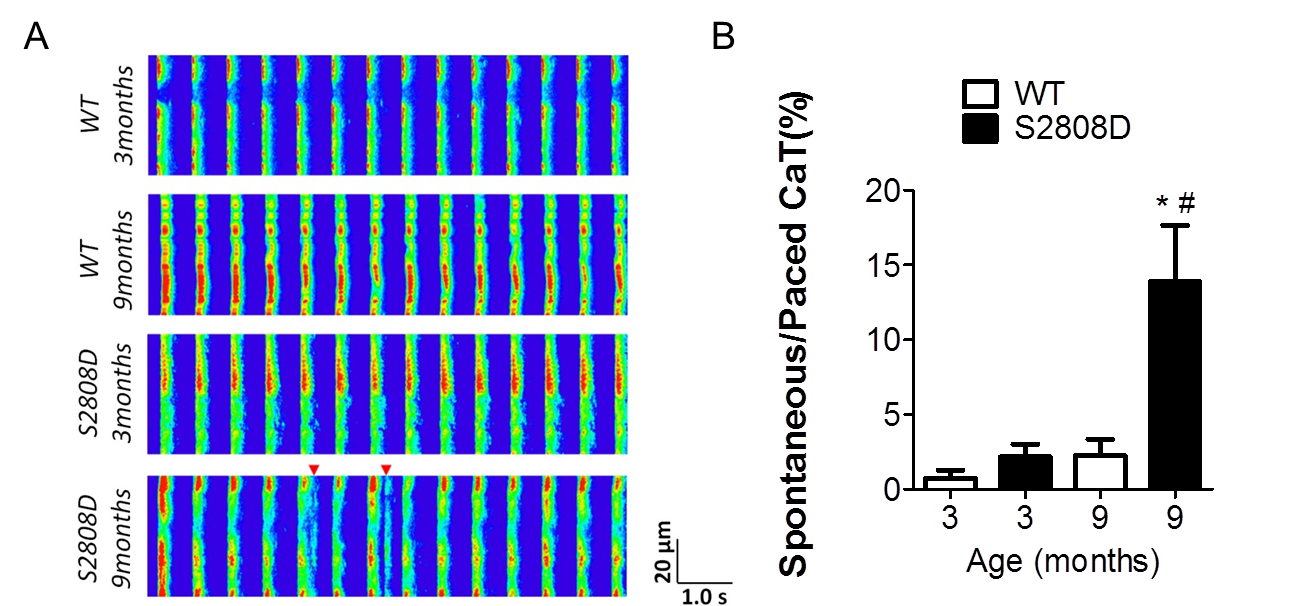


Supplementary Figure 4. Increased spontaneous Ca^2+^ transients in atrial myocytes from 9-month-old RyR2-S2808D^+/+^ mice. Linescan Ca^2+^ images (A) and quantification of Ca^2+^ transients (B). Atrial myocytes were bathed in 2 mM [Ca^2+^] imaging buffer upon 1.5 Hz-pacing during imaging. n=32~50 cells from at least 3 mice in each group. Data are shown as mean ± s.e.m. *, p<0.05 vs WT; ^#^, p<0.05 vs 3-month-old.


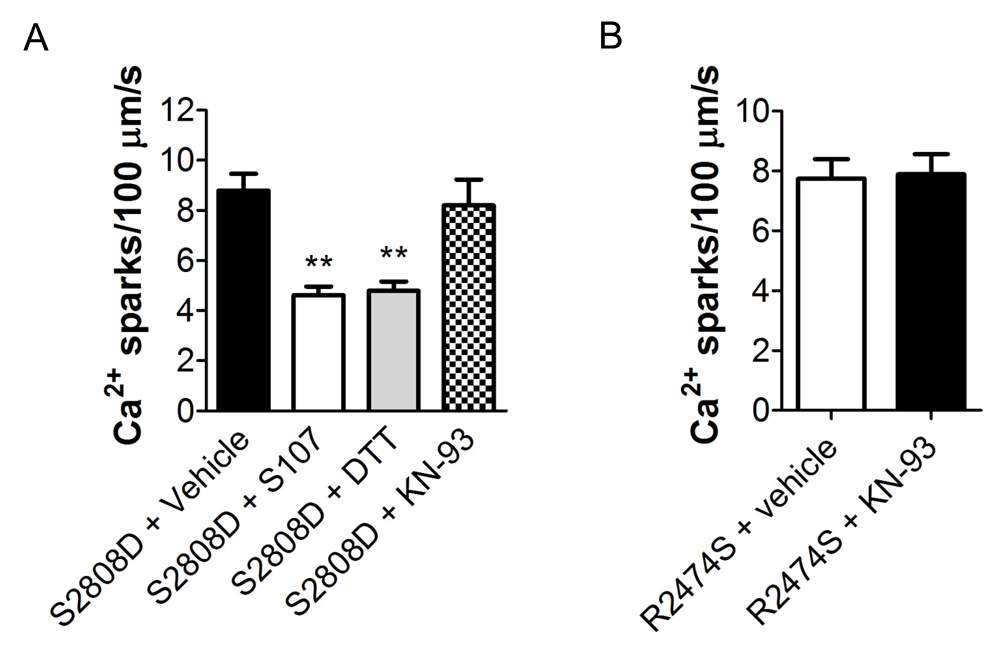


**Supplementary Figure 5**. **Increased Ca^2+^ sparks frequencies in atrial myocytes from 9-month-old RyR2-S2808D^+/+^ mice are reduced by S107 or DTT but not by KN-93.** (**A**) and 3-month-old RyR2-R2474S^+/-^ mice (**B**). Atrial myocytes were treated by S107 (10 µM), DTT (1 mM), or KN-93 (1 µM); n=33~52 cells in each group. Data are shown as mean ± s.e.m. **, p<0.01 vs vehicle.


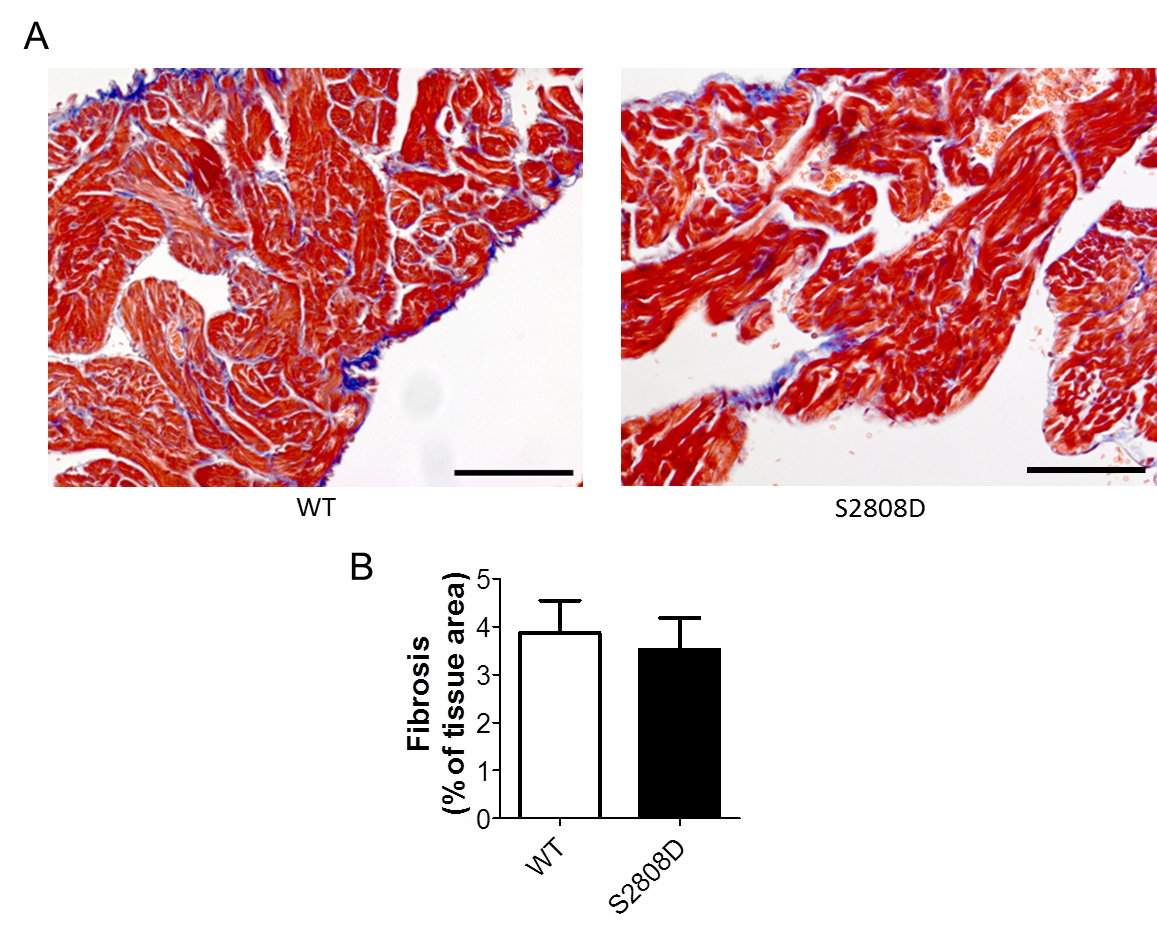


Supplementary Figure 6. Histologic examination revealed no structural differences between atria from 9-month-old WT and RyR2-S2808D^+/+^ mice.

**A**, Representative Masson’s trichrome stained sections of atria from 9-month-old WT and RyR2-S2808D^+/+^ mice; scale bar: 100 µm. **B**, Bar graph showing the quantification of fibrosis in atrial tissue. Data are shown as mean ± s.e.m.


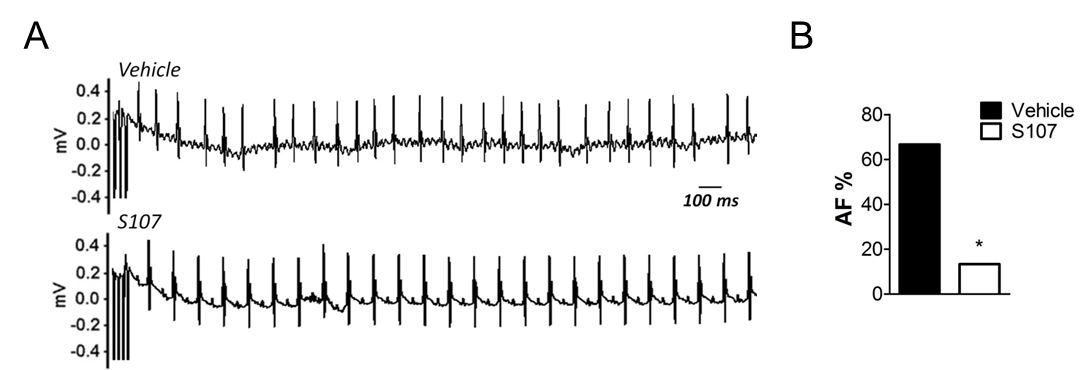


Supplementary Figure 7. Two-week treatment with S107 in the drinking water prevents AF in 9-month-old RyR2-S2808D^+/+^ mice.

**A**, Representative surface ECG traces from 9-month-old RyR2-S2808D^+/+^ mice following intra-esophageal burst pacing in vehicle (upper) and S107-treated (lower) groups. **B**, Bar graph showing the prevalence of AF following intra-esophageal burst pacing in vehicle and S107 group, n=15 mice per group. *, p<0.05 vs vehicle.


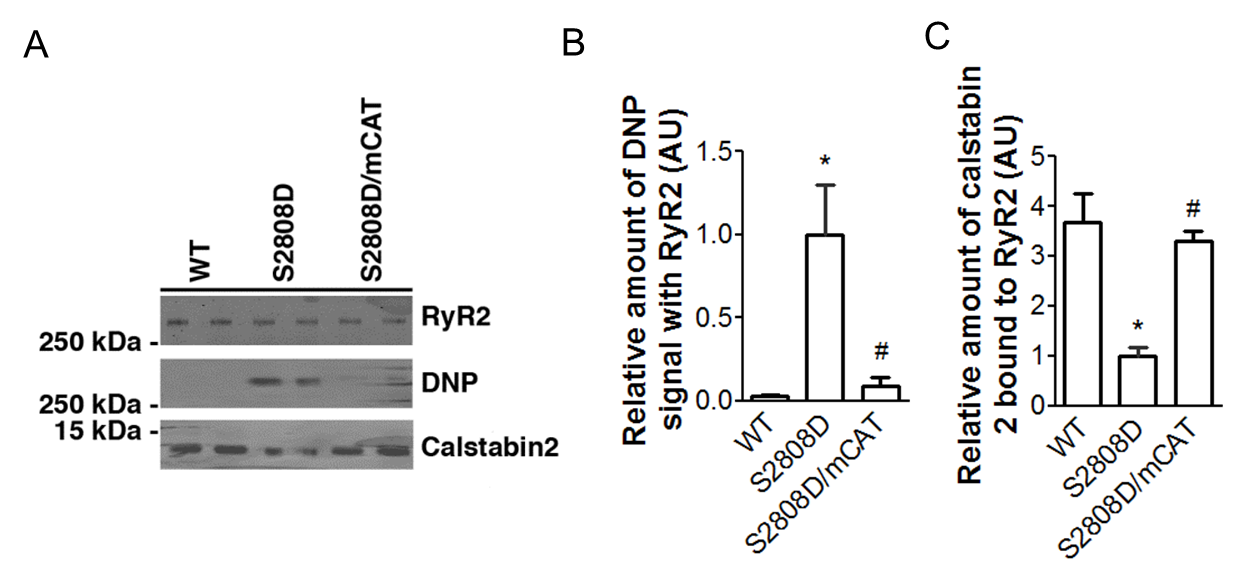


Supplementary Figure 8. A, Post-translational modifications of the RyR2 complex in atrial samples from 9-month-old WT, RyR2-S2808D^+/+^ and RyR2-S2808D^+/+^/mCAT^+^ mice. B-C, Quantification of DNP signal (B) and calstabin 2 bound to RyR2 (C); atrial samples were obtained from at least 5 mice in each group. AU: arbitrary units. All data are shown as mean ± s.e.m. *, p<0.05 vs WT; ^#^: p<0.05 vs S2808D.


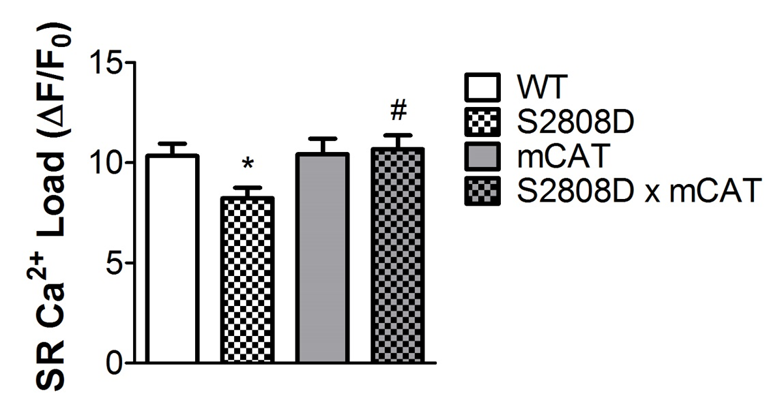


Supplementary Figure 9. Decreased SR Ca^2+^ load in atrial myocytes from 9-month-old RyR2-S2808D^+/+^,mice compared to WT mCAT and RyR2-S2808D^+/+^/mCAT^+^ mice. n=20~22 cells from ≥3 mice in each group. Data are shown as mean ± s.e.m. *, p<0.05 vs WT; ^#^, p<0.05 vs RyR2-S2808D^+/+^.


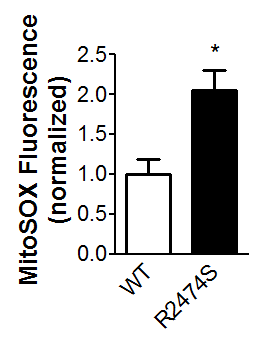


**Supplementary Figure 10**. **Mitochondrial ROS level increased in atrial myocytes from RyR2-R2474S^+/-^ mice compared to WT mice.** n=30 cells from ≥2 mice in each group. Data are shown as mean ± s.e.m. *, p<0.05 vs WT.
